# Polyamide and polyvinyl chloride microplastics induce cytotoxicity and cytokine release in primary normal human bronchial epithelial cells

**DOI:** 10.1186/s43591-026-00200-w

**Published:** 2026-05-19

**Authors:** Yvonne C. M. Staal, Irene F. Gosselink, Y. Y. Lianne Sander, Jolanda P. Vermeulen, Evert Duistermaat, Jeroen L. A. Pennings, Alexander H. V. Remels, Flemming R. Cassee

**Affiliations:** 1 National Institute for Public Health & the Environment (RIVM), Bilthoven, The Netherlands; 2 Department of Pharmacology and Toxicology, Institute of Nutrition and Translational Research in Metabolism, Maastricht University, Maastricht, the Netherlands; 3 Institute for Risk Assessment Sciences (IRAS), Utrecht University, Utrecht, The Netherlands

**Keywords:** Nanoplastics, Microplastics, Inhalation toxicology, Air-liquid interface, Quasi-ALI, Nebulization, Inflammatory response

## Abstract

Our daily and continuous exposure to airborne micro- and nanoplastics (MNPs) together with the limited information on their potential hazards, warrants the need for more information on MNP-toxicity. In this study, we investigated the effects of diverse size ranges of amorphous MNPs from environmentally relevant polymers, on Air-Liquid-Interface (ALI)-cultured Normal Human Bronchial Epithelial cells (NHBEs) by analyzing immunological response parameters 24 h after exposure. In addition, we have used this setup to compare the responses of NHBEs to MNPs using nebulization or quasi-ALI (small droplet) exposure. NHBEs responded differently to exposures of polyamide (PA) or polyvinyl chloride (PVC) particles at nominal doses between 0.003 and 0.100 µg/cm^2^. PA particles < 1 μm (but not those > 1 μm) induced dose-dependent cell death, increased IL-8 secretion and decreased MCP-1 secretion. PVC particles (< 1 μm and 1–5 μm) induced cell death at lower concentrations than PA particles. Also, an increased IL-8 secretion and decreased MCP-1 secretion was observed for PVC particles in all size fractions (< 1 μm, 1–5 μm and 5–10 μm). Comparison of nebulization versus quasi-ALI exposure indicated differences related to the exposure method, but further experimental assessment is needed for definite conclusions and to ensure that the obtained data is relevant for toxicological effects occurring in humans. Our results indicate that PA and PVC particles increase IL-8 secretion and, PA only, decreases MCP-1 secretion. It needs to be established whether these effects on cytokines also indicate an activation of immune cells.

**Supplementary Information:**

The online version contains supplementary material available at 10.1186/s43591-026-00200-w.

## Introduction

The detection of microplastics (1 μm − 5 mm) and nanoplastics (< 1 μm) in air, water and soil, and in our food and body has raised concern on their potential effects on human health [26]. Inhalation is considered one of the major exposure routes to micro- and nanoplastics (MNPs), with indoor exposure levels exceeding those outdoor and particular high levels in occupational settings (535-22531 MNPs/kgBW/day) [4]. As airborne MNPs originate from many different plastic materials, come in different shapes and sizes, may contain different intentionally or non-intentionally added chemicals and may be covered with a microbial corona, human exposure to MNPs is very diverse. This diversity makes hazard assessment of MNPs complex [27].

The majority of the toxicological studies with MNPs have been performed with spherical polystyrene (PS) particles. Since PS polymers represent only 5% of the total European plastic production, PS beads are not representative for the irregularly shaped fragments or fibers generated from the degradation of larger plastics with a diverse chemical composition (PlasticsEurope [19]). While studies applying these particles can help to identify potential hazards, there is a need for information on the effects of MNPs of environmentally relevant polymers [9]. Hazard assessment of different types of plastics particles is required to identify potential risks of exposure to the various amorphous or fibrous-shaped polymers in indoor environments, of which polyamide (PA) is predominant, with approximately 30% originating from textiles [3, 28]. Especially insight into toxicological responses related to polymer type, particle size and particle shape are important for risk assessment of MNPs.

Primary normal human bronchial epithelial cells (NHBEs) are a widely used model for studying respiratory diseases and barrier function. Derived from the epithelial lining of the bronchi, NHBEs provide a physiologically accurate representation of the bronchial epithelial barrier. NHBEs can be cultured at air liquid interface (ALI), creating an environment where the cells are in contact with the air while nutrients are supplied from the basolateral side [20]. This culture method allows for cellular differentiation and facilitates crucial features of the respiratory epithelial barrier, including ciliary function and mucus production [20]. In addition, it allows the cells to be exposed to airborne substances, such as MNPs.

Several methods can be used to expose ALI-cultured cells [22]. The most representative method for aerosol exposure is a continuous flow system, in which the cells are exposed to a continuous flow of particles similar to the human in vivo situation. Continuous flow exposure however, generally requires relatively large quantities of test substance. To obtain MNPs that resemble the environmental degradation process, mechanical fragmentation was used by the MOMENTUM consortium. Though this method results in particles in a suspension that also contains chemicals that are released during the process [18], though quantities are limited. To distinguish between particle effects and chemical effects, we have therefore used this solvent without particles (referred to as leachates). When limited quantities of material are available, exposure by nebulization of the suspension above the cells is a good approach to reach a uniform deposition on the cells. Although this is an efficient way of dealing with low quantities, a substantial part will not reach the cells as it is equally deposited on the total surface area of the device. The application of a small volume of a suspension directly onto the cells (also referred to as quasi-ALI exposure) can be used as an alternative, without significant losses of material [8, 13]. Although direct comparisons of both exposure methods are not described in the literature, it is conceivable that differences in exposure method also lead to differences in the outcomes. For example, exposure by nebulization results in a uniform deposition on the surface area of the insert, whereas quasi-ALI exposure results in a high local dose at the site of application. Also, for quasi-ALI exposure the particles are applied in suspension, whereas nebulization leads to deposition of small droplets with particles. The choice of the exposure method may impact the biological responses, which is the reason to compare nebulization with quasi-ALI exposure in our experiments.

It has been noted that exposure to smaller-sized PS of PA particles (< 1 μm) had a more detrimental effect on cell viability of A549 and BEAS2B cells when compared to exposure to larger MNPs (> 5 μm) when using mass as a comparative exposure metric [6, 7]. Additionally, decreasing particle sizes are associated with higher surface-to-volume ratios. This implies that, at the same mass, smaller particles possess a greater total surface area available for interaction with biological tissues compared to larger particles. In addition, larger particles are also more rapidly cleared by the mucociliary clearance system [10].

The aim of our research was to assess the inflammatory response (cytokine production) of NHBEs after exposure to various sizes of two types of MNPs. Cell viability and cytokine/chemokine responses of NHBEs were determined after exposure to different sizes of PA and polyvinyl chloride (PVC) using aerosol exposure. In addition, cultures were exposed to leachates from MNPs, to distinguish chemical effects from particle effects. We hypothesized that smaller-sized MNPs would induce a larger toxicological response on NHBEs compared to larger particles, as the total number of particles at the same mass-based dose is much higher for smaller-sized particles. Subsequently, to evaluate the role of the exposure method, we have used this setup to compare the responses of NHBEs to MNPs using nebulization or quasi-ALI exposure.

## Materials and Methods

### Materials

PVC and PA microplastic particles, size ranges of < 1 μm, 1–5 μm and 5–10 μm and suspended in 1-propanol, were supplied by TNO [17, 18]. In short, initial size reduction of commercial powders or pellets to 500 μm was performed using a centrifugal mill under cryogenic conditions. Further ball-milling in 1-propanol was performed to reach the final particle sizes. Fractionation into size ranges of < 1, 1–5, 5–10 μm was performed by sedimentation and filtration, resulting in 20 mg/ml suspensions. These suspensions were first diluted in 1-propanol to 1 mg/ml and further diluted to obtain the concentrations used for nebulization (see below), while vortexing in between. To distinguish between particle- and chemical toxicity, the leachate of PVC and PA MNPs was derived during the production process, and consists of 1-propanol, which may contain chemicals leaking during production and milling. Leachates are the same for each particle size fraction, but different for PA and for PVC. Leachates were similarly diluted in 1-propanol and served as a control, in addition to a 1-propanol vehicle control. In all experiments, an unexposed incubator control (IC) was included.

### Cell culturing

NHBEs were obtained from four different donors (Lonza, Walkersville, MD, USA), see Table 1.

**Table 1:** Donor numbers and characteristics

Number	Donor	Sex	Age (years)
1	18TL215671	Female	47
2	0000613375	Female	65
3	0000627466	Female	24
4	0000580582	Male	57

First, cells from donors 1, 2 and 3 were separately cultured in a flask with PneumaCult Ex Plus basal medium (Stemcell Technologies, Seattle, WA, USA) supplemented with PneumaCult Ex Plus 50x supplement (Stemcell Technologies), 1% Penicillin (10000 U/mL) and Streptomycin (10000 U/mL) (Gibco, Paisley, Scotland), and 0.5% hydrocortisone stock solution (96 µg/ml) (Stemcell Technologies). Cultures were incubated at 37 °C with 5% CO_2_ and refreshed every other day. Upon 80–90% confluency, cells were washed with Hank’s balanced Salt Solution (HBSS) (Gibco), rinsed with HEPES Buffered Saline Solution (HEPES-BSS, Lonza) and subsequently harvested with trypsin-EDTA (Lonza). Cells of donors 1, 2 and 3 were pooled and seeded on the apical side of collagen-coated transwell inserts (Corning® 24 mm Transwell® with 0.4 μm Pore Polyester Membrane Insert), with a total density of 100,000 cells/insert in Pneumacult Ex Plus medium. PneumaCult Ex Plus medium was also added to the basolateral chamber of the well. Cells were refreshed for both chambers with PneumaCult Ex plus medium every other day. When confluency reached 90%, cells were airlifted by removing the culture medium from the apical side. Subsequently, only the basolateral chamber of the well was refreshed with PneumaCult ALI Basal medium (Stemcell Technologies) supplemented with PneumaCultALI 10x supplement (Stemcell Technologies), PneumaCult-ALI Maintenance supplement (Stemcell Technologies), 1% Penicillin (10000 U/mL) and Streptomycin (10000 U/mL) (Gibco), 0.5% hydrocortisone stock solution (96 µg/ml) (Stemcell Technologies) and 0.2% Heparin Solution (Stemcell Technologies). Cells were kept at ALI at 37 °C with 5% CO_2_, while refreshing basolateral medium three times a week. Mucus was removed by washing the cells on the apical side once a week with HBSS buffer (Gibco, 14065056) diluted in ultra-pure water (Braun’s water, 14269716). The cultured cells were used for exposure 4 or 5 weeks after airlift. A second experiment, described in more detail in supplementary file 1, was conducted to compare exposure methods. For this, cells from three different donors 1, 3 and 4 (Table 1) were (separately) cultured on inserts. Other than that, the same cell culture procedure was followed as described above.

One day before exposure, the basolateral chamber of the well was refreshed with PneumaCult ALI medium without supplements of hydrocortisone, penicillin/streptomycin and heparin. Hydrocortisone and heparin possess anti-inflammatory properties, which could potentially inhibit exposure responses, given that the research focused on cytokine production by NHBEs exposed to MNPs.

### Exposure of epithelial cells to airborne MNPs

To expose the cells, we have used a radial in vitro aerosol exposure system (RIVAES) which was designed and made at RIVM [12] and inspired by the design of the VITROCELL® Cloud exposure system (VitroCell, Waldkirch, Germany). In this exposure system, the transwell inserts are placed radially to minimize variation in deposition. The system has a slightly smaller surface area than the VITROCELL® Cloud system, resulting in a slightly higher deposition. Details on the deposition and the variation in deposition can be found in Supplementary file 2. It is equipped with a refined temperature control system resulting in a stable temperature at the transwell inserts. The system was equipped with a nebulizer to generate aerosols for microplastic exposures. Prior to the start of the exposure, the exposure system and glass chamber were cleaned and warmed to 37 °C. A nebulizer for large particles (9–12 μm) (H-T45 Electronic sprayer, Tekceleo) was set to a flow rate of 25%. Concentrations of the nebulized suspensions were 60 µg/ml, 10 µg/ml and 2 µg/ml for the high, mid and low dose respectively (all in 100% 1-propanol, without Phosphate-buffered saline (PBS) or BSA). A volume of 200 µl was nebulized for each exposure. Taking into account the nebulized volume, working concentrations and total horizontal surface are of the exposure system (120.8 cm^2^), a cell growth surface area of 1.12 cm^2^ per insert the cultures were exposed to nominal doses of 0.100 µg/cm^2^, 0.017 µg/cm^2^ or 0.003 µg/cm^2^. For this calculation the losses onto the vertical walls of the RIVAES were considered negligible, whereas losses would result in even lower deposited dose values. The actual deposited dose could not be assessed using the Quartz Crystal Microbalance (sQCM 12, Vitrocell, Waldkirch, Germany) that is positioned at one of the positions of the RIVAES due to the limit of detection which is ≥ 100 ng/cm^2^,. However the sQCM was used to monitor when a steady state was reached after the nebulization and subsequent evaporation of solvent due to the temperature gradient (21 versus 37 °C, the time at which the inserts with the cells were moved to a new 12-wells plate, with fresh medium at the basolateral side and returned to the incubator (37 °C with 5% CO_2_) for 24 h until sampling. Exposures were performed in multiple runs with *n* = 3 or 4 per run (Table 2), on a total of 6 different days using three batches of cell cultures. The total number of replicate inserts was 12 (PA solvent controls), 8 (PA leachate/particles), 11 (PVC solvent controls) or 7 (PVC).

**Table 2:** Overview of the exposures, including the exposure day and cell culture batch, indicating the number of insert used for each exposure and condition

	number of inserts per concentration					
Cell culture batch	1	1	2	2	2	3
Exposure day (run)	1	2	3	4	5	6
Solvent control*	4	4	4	4	3	4
PVC leachate*	4				3	
PVC < 1 μm*		4			3	
PVC 1–5 μm*		4			3	
PVC 5–10 μm*		4			3	
PA leachate*	4			4		4
PA < 1 μm*			4			4
PA 1–5 μm*			4			4
PA 5–10 μm*			4			4

* number of inserts are for one concentration, for every experiment, the three concentrations are tested simultaneously

### Particle size distribution

Particle size distribution of the suspensions was determined before and after nebulization by placing the nebulizer on top of a 50 ml tube. The MNP suspension was 10 times diluted in 1-propanol and particle sizes were measured before and after nebulization. Nebulization resulted in tiny droplets were impacted in this 50 mL tube until a sufficient amount of suspension was formed to be analyzed. Particle size distribution was measured using a Particle size analyzer (Disc Centrifuge 24000, CPS Instruments, Prairieville, LA, USA), and a sucrose gradient of 8–24%. Calibration was done using particles with a size of 1.30 and 0.544 μm. These measurements were not conducted for PVC 5–10 μm particles.

### Transepithelial electrical resistance for cell membrane integrity

The integrity of cellular barriers was measured before exposure by determining the transepithelial electrical resistance (TEER) using a Milli cell ERS-2 and a chopstick with electrodes, 24 h after exposure, as previously described [23]. Measurements using chopsticks tend to be very variable and dependent on the location of the chopsticks in the insert and therefore not sensitive enough to detect small changes in barrier integrity related to exposure. These data were used to confirm that the cell cultures had developed a barrier and were of good quality for exposures. All TEER values were above 600 Ω/cm^2^ indicating a proper barrier function.

### Cytotoxicity

To analyze cytotoxicity, an Lactate Dehydrogenase (LDH) assay was conducted 24 h after exposure using the basolateral medium. A cytotoxicity Detection kit (Roche, 11644793001) was used as previously described [23].

For the PA and PVC nebulization experiment, the LDH response was compared to the LDH response of lysed cells (LDHmax). A relative cytotoxic percentage exceeding 10% is considered indicative of cytotoxicity. As some LDH response is expected due to cell turnover, this cut-off is used to discriminate normal cell turnover from cytotoxicity, as also used previously [23]. For the comparison between quasi-ALI and nebulization we have used a standard curve to quantify LDH response.

### Cytokine and chemokine responses

Basolateral medium of exposed NHBEs was used for the quantification of cytokines and chemokines. The basolateral medium was specifically selected, as this is more relevant than the apical side for potential interaction with (systemic) immune cells and NHBE cells have been shown to secrete more IL-8 in the basolateral compartment [8]. The LEGENDplex bead-based immunoassay, specifically the HU Essential immune response panel (13-plex) (Lot no. B338755) from BioLegend, was used as described previously [23]. The panel includes the following cytokines: Interleukin-4 (IL-4), Interleukin-2 (IL-2), C-X-C motif chemokine 10 (CXCL10, or Interferon-gamma-induced protein 10 (IP-10), Interleukin-1beta (IL-1β), Tumor Necrosis Factor alpha (TNF-α), Chemokine (C-C motif) ligand 2 (CCL2, or Monocyte chemotactic protein 1 (MCP-1), Interleukin 17 A (IL-17 A), Interleukin 6 (IL-6), Interleukin 10 (IL-10), Interferon gamma (IFN-γ), Interleukin 12p70 (IL-12p70), C-X-C motif chemokine ligand 8 (CXCL8, or interleukin 8 (IL-8) and Transforming Growth Factor, Beta 1 (TGF-β1, Free Active Form).

### Real-time quantitative-PCR

For gene expression analysis, cells were washed with PBS and directly lysed using QIAzol (Qiagen). RNA was isolated as previously described [7]. Subsequently, 400 ng RNA was transformed to cDNA, and Real-time quantitative PCR (qPCR) amplification was performed as previously described [7].

QPCR data were analyzed using the CFX Maestro software (Bio-Rad) and LinRegPCR software 2014. The gene expression of *C-X-C* motif chemokine ligand 1 (*CXCL1*, or growth-regulated oncogene-alpha *(GROα)*, C-X-C motif chemokine ligand 2 (*CXCL2*, or Macrophage inflammatory protein 2-alpha *(MIP2α)* and C-X-C motif chemokine ligand 8 (*CXCL8*, or interleukin 8 (IL-8)) was normalized based on the expression of four housekeeping genes (beta actin (*ACTB*), beta-2-microglobulin (*B2M*), cyclophilin A (*PPIA*), and ribosomal protein L13a (*RPL13A*)). Normalized gene expression levels in exposed conditions were compared with corresponding solvent control, whereafter the effects of the exposure method and BSA were analyzed.

### Statistical analysis

MS Excel was used for data collection and processing. Statistical analyses were performed in R statistical software (version 4.4.0).

For statistical analysis of cytokine data, data for each insert were log-transformed followed by a two-way ANOVA to test for effects of exposure (versus 1-propanol as carrier control), with the cell culture batch as covariate to allow for inter-batch variation. The R code for the statistical test was as follows: *glm(log_il8 ~ experiment + exposure*,* data = mydata)*, where *log_il8* denotes the log-transformed IL-8 concentration (or, where applicable, the log-transformed MCP-1 concentration or the log-transformed LDH response); *experiment* denotes the cell culture batch as a categorical variable; *exposure* denotes a categorical variable indicating one of the 14 exposure scenarios (e.g. PA_leachate_0.003 µg or PVC_5–10µm_0.017 µg, see Fig. 3); and *mydata* either comprises the PA or PVC data plus concurrent controls. Each exposure was considered as a separate categorical variable, to avoid introducing assumptions about size and dose additivity. This was done for PA and PVC separately. P-values were corrected for multiple testing using the Benjamini-Hochberg false discovery rate.

For comparison between nebulization and quasi-ALI exposure, a similar approach was followed after checking for interaction terms (particle: exposure method and particle: BSA) which were found not to contribute. The final ANOVA model included particle exposure, nebulization/quasi-ALI and presence/absence of BSA as independent variables and donor as a covariable. The R code for the statistical test was as follows: *glm(log_il8 ~ pvc_yn + cloud_yn + bsa_yn + donor_id*,* data = mydata)*, where *log_il8* denotes the log-transformed ratio of the IL-8 concentration to the donor-matched average of quasi ALI vehicle controls (note that IL-8 can be substituted by one of the other parameters shown in Figure S1.1 and S1.2); *pvc_yn* denotes the presence (1) or absence (0) of PVC; *cloud_yn* denotes nebulization (1) or quasi-ALI (0); *bsa_yn* denotes the presence (1) or absence (0) of BSA; and *donor_id* is a categorical covariable that indicates the donor.

## Results

### Effects of nebulized PA and PVC particles

#### Particle size distributions before and after nebulization

Particle size distribution curves of the MNPs suspensions after nebulization were generally similar to those before nebulization. Still, for the samples that contained the larger particles the average particle size was only slightly lower after nebulization (Table 3) which indicates that only the largest particles may not be able to pass the mesh of the nebulizer and most of the particles deposited onto the cells.

**Table 3:** Median particle size for PVC and PA particles before and after nebulization in 1-propanol

Sample	Size on label (µm)	Median number size before nebulization (µm) (CV(%))	Median number size after nebulization (µm) (CV(%))
PVC	< 1	0.14 (105.5%)	0.14 (89.0%)
PVC	1–5	2.88 (44.4%)	2.01 (41.6%)
PA	< 1	0.15 (73.0%)	0.15 (57.7%)
PA	1–5	1.14 (52.5%)	0.90 (41.7%)
PA	5–10	0.99 (54.6%)	0.75 (34.7%)

PVC 5–10 μm particles were not measured. Particle size distribution curves can be found in the Supplementary file 3

### Cytotoxicity

**Fig. 1:**
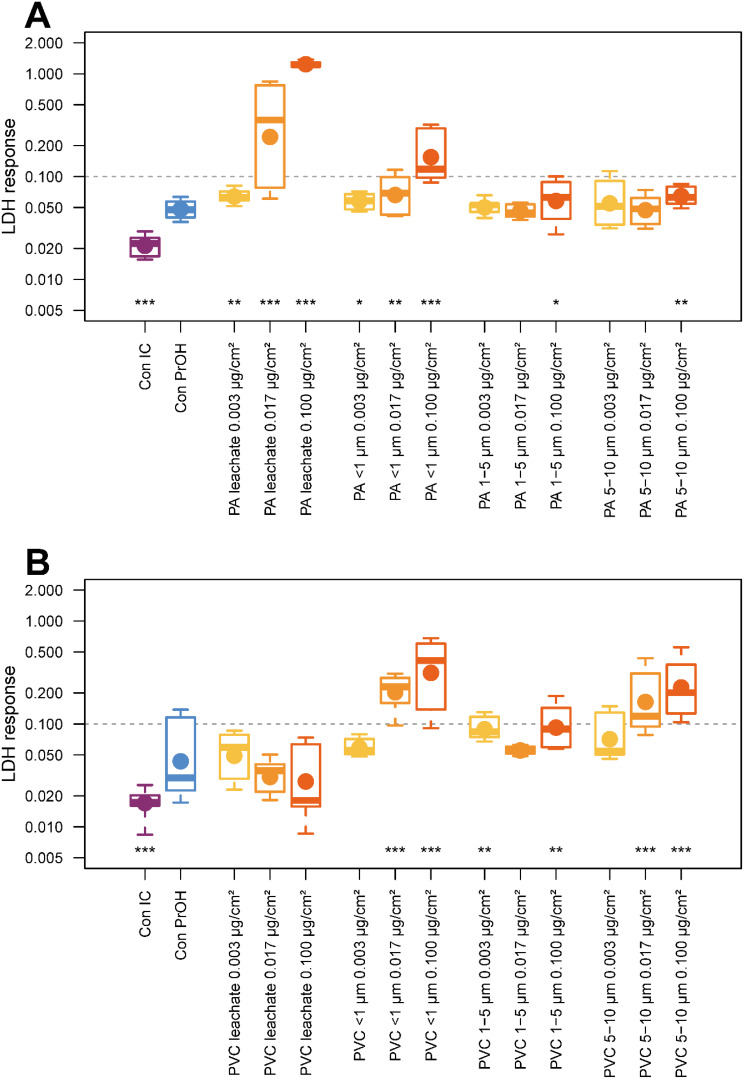
LDH response, relative to the maximum LDH response of lysed cells (set to 1), of NHBE cells exposed via nebulization to PA (**A**), PVC (**B**) and their leachates or vehicle control, 24 h after exposure. Responses are relative to the maximum LDH response whereas the y-axis follows a log scale. Data of two independent experiments for PA and PVC each with three or four inserts per condition. Leachates are expressed in µg/cm^2^, which correspond to the same dilutions as the particles even though no particles are present in the leachates. The box indicates the interquartile range, whiskers indicate the maximum-minimum range, the line in the box indicates the median, and the filled circle indicates the geometric mean. The dashed line indicates the 10% cytotoxicity level. Significant differences with the vehicle control (Con PrOH) are indicated with asterisks: * = *p* < 0.05; ** = *p* < 0.01; *** = *p* < 0.001

Cytoxicity to NHBE cultures, established by pooling cells from three donors, in response to PA of PVC exposure was determined by measuring the LDH leakage in the basolateral compartment 24 h after exposure. Figure 1 shows the cytotoxicity of NHBEs exposed to PA or PVC particles of different sizes 24 h after exposure relative to the maximum LDH response. A comparison with a 10% of the maximum response was made, as statistical analysis of the data, although important, at low levels of cytotoxicity mainly indicates low variation in the response and normal cell turnover instead of being an indicator for cytotoxicity. PA leachate induced a dose-dependent increase in cytotoxicity compared to the vehicle control (Fig. 1A). A similar dose-dependent increase in loss of cell viability was found for PA < 1 μm particles, although the highest dose of this particle exposure was less cytotoxic than the leachate. LDH release of cells exposed to PA particles of larger sizes was below 10% of the maximum response, indicating that limited cytotoxicity occurred. PVC leachate did not affect cell viability (Fig. 1B). However, PVC < 1 μm, PVC 1–5 μm and PVC 5–10 μm particles slightly induced a loss of cell viability, which was dose-related for the smallest and largest particles. Although statistically significant, LDH release was still relatively low compared to the maximum response.

**Fig. 2:**
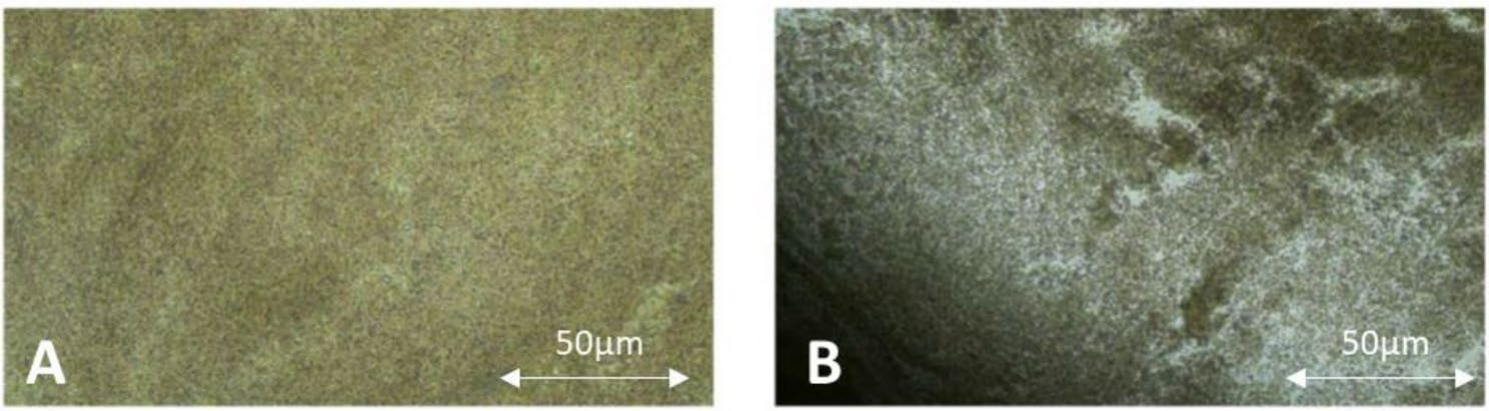
Microscopic images (40x magnification) of NHBEs, the incubator control (**A**) and NHBEs exposed to the highest dose of PA leachate (**B**), both 24 h after exposure

Microscopic observation of cells exposed to the highest dose of PA leachate confirmed these effects (Fig. 2) and revealed abnormal cell morphology and cell membrane disruption (Fig. 2B). The other exposures did not result in such effects on cell morphology.

### Cytokine and chemokine responses

**Fig. 3:**
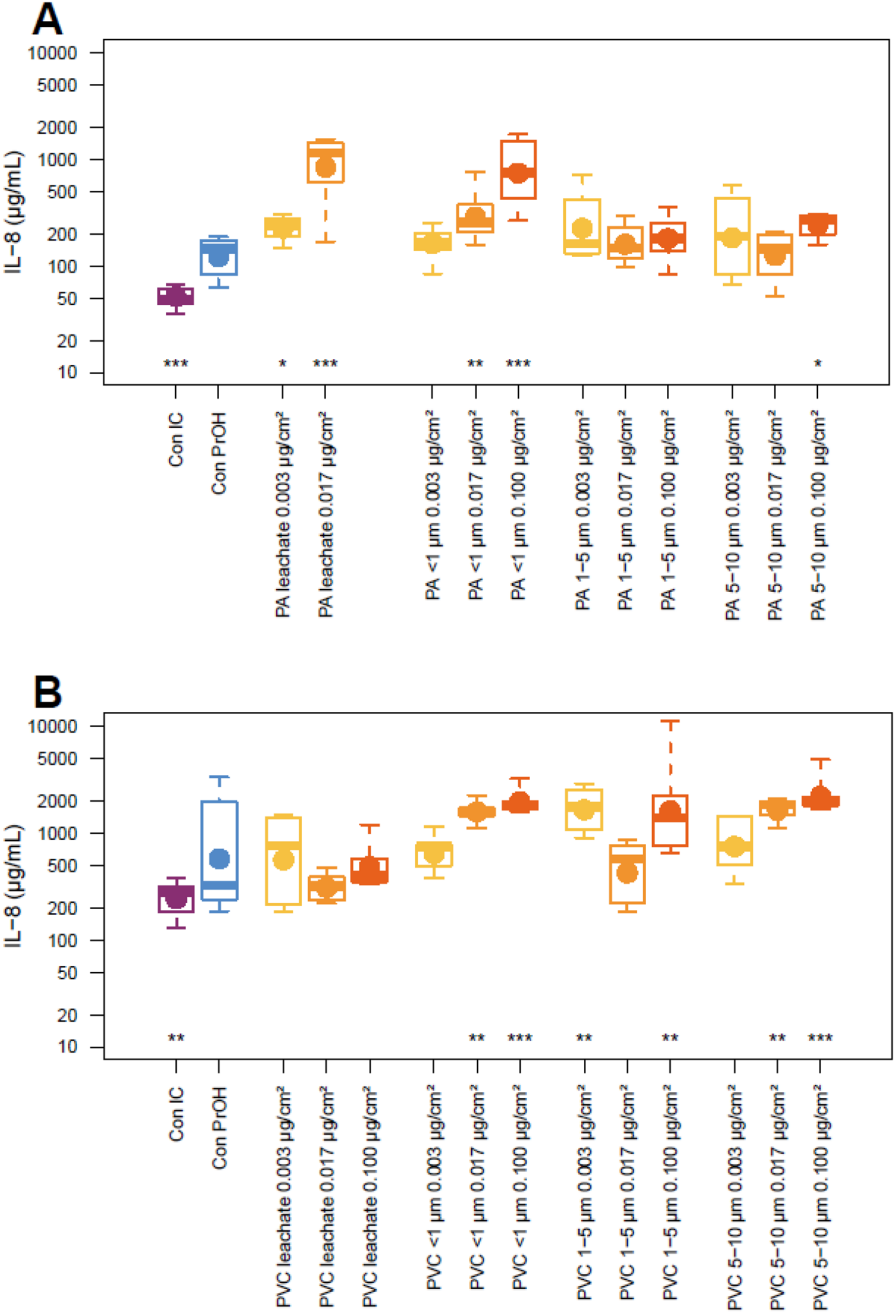
IL-8 responses of NHBEs 24 h after exposure to PA (**A**) or PVC (**B**) particles as measured in basolateral medium. Differences were evaluated compared to the vehicle control (solvent control, 1-propanol, ConPrOH). Data of two independent experiments each with three or four inserts per condition. Leachates are expressed in µg/cm^2^, which correspond to the same dilutions as the particles even though no particles are present in the leachates. The highest dose of PA leachate was cytotoxic and was therefore not included in further evaluations. The box indicates the interquartile range, whiskers indicate the maximum-minimum range, the line in the box indicates the median and the circle indicates the geometric mean. Asterisks (*) indicate significant differences (compared to vehicle control (1-propanol, Con PrOH), as follows * = *p* < 0.05; ** = *p* < 0.01; *** = *p* < 0.001

**Fig. 4:**
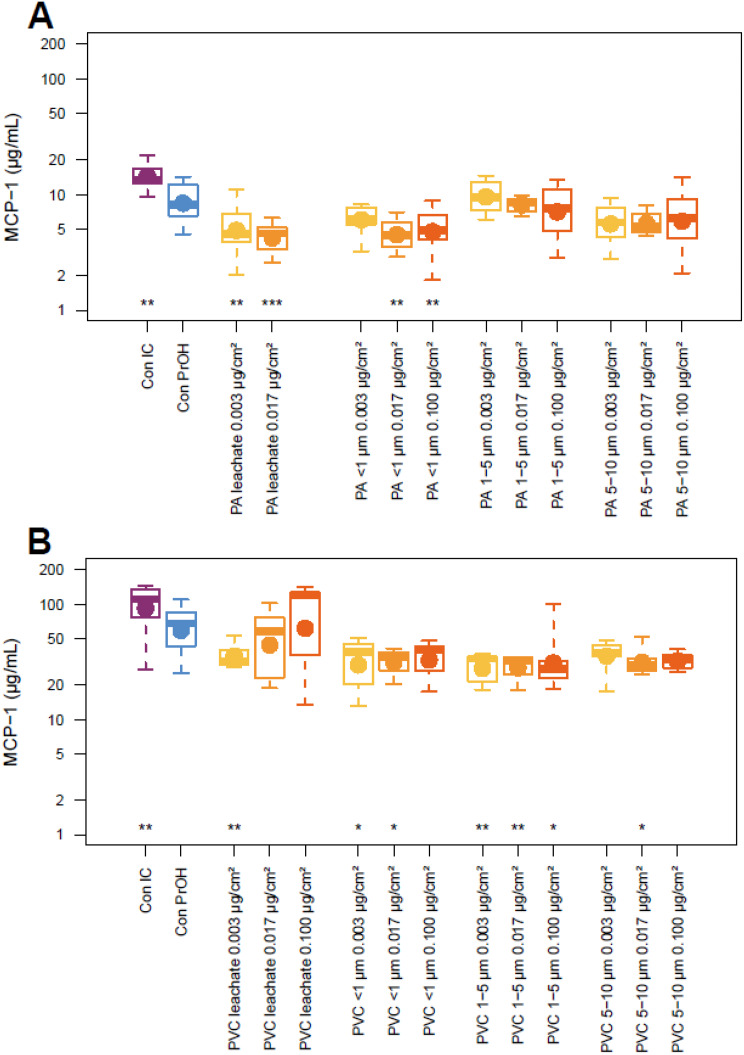
MCP-1 responses of NHBEs 24 h after exposure to PA (**A**) or PVC (**B**) particles in basolateral medium. Differences were evaluated compared to the vehicle control (solvent control, 1-propanol). Data of two independent experiments each with three or four inserts per condition. Leachates are expressed in µg/cm^2^, which correspond to the same dilutions as the particles even though no particles are present in the leachates. The highest dose of PA leachate was cytotoxic and is therefore not included in further evaluations. The box indicates the interquartile range, whiskers indicate the maximum-minimum range, the line in the box indicates the median and the circle indicates the geometric mean. Asterisks indicate significant differences compared to solvent control (1-propanol), significance is indicated as follows: * = *p* < 0.05; ** = *p* < 0.01; *** = *p* < 0.001

Of the 13 cytokines and chemokines measured, only IL-8 and MCP-1 were affected by the exposures (Figs. 3 and 4). Other cytokines, IP-10, IL-4, IL-2 and IL1β were also measured above detection limits, but those were not affected by the exposure and therefore not further discussed.

Exposure to PA leachate resulted in a statistically increased production of IL-8 and decreased production of MCP-1 at the low and mid concentration. Considering that the highest concentration of PA leachate induced cytotoxicity, and the mid concentration to a lesser extent, the effect at the lowest concentration would only be indicative of an effect on IL-8 and MCP-1 release. Exposure to PA < 1 μm particles resulted in statistically significantly higher IL-8 production and a lower MCP-1 production in a dose-dependent manner. Again here, the highest concentration also induced cytotoxicity. The larger PA particles (1–5 μm and 5–10 μm) did not affect IL-8 or MCP-1 production, except for a slight statistically significant increase in IL-8 for PA 5–10 μm.

Exposure to PVC particles of all three sizes resulted in increased IL-8 secretion and decreased MCP-1 secretion (although not statistically significant for all concentrations and all sizes). In contrast to PA leachates, exposure to PVC leachates did not result in effects on IL-8 or MCP-1 secretion. The significant reduction in MCP-1 at the lowest concentration of PVC leachate is likely due to a low variation compared to the other conditions. Exposure to the PVC particles < 1 μm and 5–10 μm resulted in a dose dependent decrease in IL-8 secretion. Effects on IL-8 after exposure to PVC 1–5 μm particles are significant, but not dose-related. Exposure to PVC leachates or particles did result in significantly different MCP-1 excretion levels for PVC leachate (low concentration), <1 μm particles (low and mid concentration), 1–5 μm particles (all concentrations) and 5–10 μm particles (mid concentration), but none of these was dose dependent.

## Discussion

The objective of our experiments was to investigate the effects of environmentally relevant MNPs on NHBEs by analyzing immunological response parameters. Summarizing, we observed differences in effects between PA and PVC particles. PA particles < 1 μm induced dose-dependent effects on cell death, increased IL-8 secretion and decreased MCP-1 secretion, which was also found for the leachates. It therefore seems that components in the leachate are responsible for this effect. Since the chemical composition of the leachate is unknown, it is not possible to relate this effect to a specific constituent. PVC particles (< 1 μm, 1–5 μm and 5–10 μm) exposure resulted in slightly damaged cells. Also, an increased IL-8 secretion and decreased MCP-1 secretion was observed for PVC particles in all size fractions (< 1 μm, 1–5 μm and 5–10 μm), but not for the PVC leachate. We only found exposure related effects on IL-8 and MCP-1, although NBHEs have been shown to produce other cytokines and chemokines as well [15]. The hypothesis was that smaller-sized MNPs would have a greater impact on NHBE’s compared to larger-sized particles at equal mass concentrations, which was confirmed for PA, but not for PVC MNPs.

Although the diversity in MNPs is too large to be covered in a single experiment, our results help to understand the biological responses of MNPs. Especially important is that the MNPs used were obtained by milling and grinding larger particles, thereby resulting in particles of different (and irregular) shapes and sizes, which can be considered more environmentally relevant then manufactured MNPs [7].

Hazard assessment of MNPs, with other particles than spherical polystyrene particles is very limited, especially with respect to respiratory effects studied under physiologically relevant conditions, like ALI exposure. Garcia-Rodriguez et al., assessed the effects of polylactic acid nanoparticles (average 130 nm) using quasi-ALI exposure of Calu-3 cells [5]. Although their applied (nominal) doses were higher than in our study (2.5–20 µg/cm^2^, versus 0.003–0.100 µg/cm^2^ in our experiment), they also found effects on cytokine production and genotoxic effects. Vailionyte et al., exposed submerged-cultured BEAS-2B cells to environmentally relevant low-density polyethylene (LDPE) particles (10–1000 µg/cm^2^, median diameter of 566 nm) and also found an increased secretion of proinflammatory mediators (CD62E, CD62P, ICAM-1, IL-6, IL-8) in addition to, among others, effects on oxidative stress and cell proliferation [24]. PA fibers were also found to induce IL-8 secretion in nasal cells cocultured with macrophages [16]. However, these effects on cytokine/chemokine secretion were not consistently found in all in vitro experiments, using macrophages or airlifted cultures of BEAS-2B cells, nor for the same particles [8, 25], suggesting that polymer type and shape, cell type or possibly exposure method may play a role in the detection of inflammatory responses. Specifically for PVC, inflammatory responses were found in cell models for other organs, such as the intestine and blood [2, 11], in line with the potential effect of PVC on cytokine production we found in our experiments.

Most research on MNPs focusses on the particles themselves. Our experimental data indicate that not just the particles, but also the chemicals leaching from the plastic material may be harmful for human tissues, which was also observed for lung organoids previously [21]. PA leachates induced cytotoxicity and cytokine responses at lower doses than PVC leachates, which warrants identification of the chemical composition of these leachates that may explain differences in biological effects.

The doses in our nebulization exposure experiment were substantially lower than those used by others for submerged and quasi-ALI exposures [8]. Effects were observed at nominal doses of 0.100 µg/cm^2^, 0.017 µg/cm^2^ or 0.003 µg/cm^2^, for the high, mid and low dose respectively, which corresponds to concentrations of 60 µg/ml, 10 µg/ml and 2 µg/ml for submerged conditions. The fact that we see responses at much lower doses using nebulization exposures compared to quasi-ALI exposure, and also at lower concentrations then previously found with quasi-ALI exposure [5, 16, 24], may be explained by protein corona formation in cell culture medium, which plays an important role in toxicological responses of MNPs [14]. A protein corona on particles may prevent direct contact between the particles and the cells, which may impact biological responses.

Unfortunately, these low doses were too low to be measurable by the QCM and therefore, the actual deposited dose could not be determined. However, as we found effects on the cells upon exposure, we can assume that the cells were actually exposed. As dosing was based on nominal dose, e.g. particle mass nebulized, assuming an equal distribution of the particles over the surface area, the actual dose could have been lower. Larger particles were retained in the nebulizer, and although this could not be confirmed with particle size measurements (differences before and after nebulization were small), this may have resulted in lower dose then calculated and a shift towards smaller particles. In addition, potential losses due to deposition on the chamber walls may have occurred, or by non-uniform deposition, the dose may have been different between inserts. All together, we can assume that the nominal dose is more likely to be an over estimate of the actual dose then an underestimate.

For risk assessment, it is important to evaluate the exposure concentrations with the concentrations found in ambient air. Based on the surface area of the insert and the gas exchange area of the lung (70-140m^2^) [1], the total inhaled particle mass can be calculated. For our highest nominal dose (0.1ug/cm^2^) this corresponds to 70-140 mg deposition. Estimation of the deposition in the tracheobronchial and pulmonary region of particles with an MMAD of 5 μm and a GSD of 1, including clearance using the MPPD model, gives an estimated deposition in the bronchial and pulmonary regions of about 20%. It should be noted that particles with a low density, like MNPs, are outside the applicability domain of the MPPD model, A nominal dose of 70–140 mg corresponds therefore to a total estimated quantity of 350-700 mg inhaled. Based on the density and size of the particles, this corresponds to approximately 4 to 9 × 10^9^ particles in total. With a tidal volume of 625 ml and a breathing frequency of 12 per minute, assuming a 24 h exposure period, the particle concentration would be 300–800 particles per m^3^ (based on nominal doses) This is in the range found for indoor particle concentrations [29].

NHBE cells from three different donors were pooled to represent an average response in the measurements in contrast to donor differences in effect. This approach has pros and cons. Among the pros are a more consistent response across experiments, with only the difference in exposure moment and cell culture compared to the exposure of individual donors at different moments which does not allow discrimination between exposure condition effect and donor effect. A disadvantage is that the effects of individual donors cannot be evaluated. The results in supplementary file 1, showing that the main contributor of the effects is the variation in donor, indicates that donor differences should not be neglected. With only three donors in one experiment, evaluation of donor-dependent effects would, however, not be possible.

## Conclusion

Our results indicate that PA and PVC particles increase IL-8 secretion and, PA only, decreases MCP-1 secretion. It needs to be established whether these effects on cytokines also indicate an activation of immune cells. Also, as PA leachates induced cytotoxicity and, at lower concentration, effects on secretion of IL-8 and MCP-1, as the chemical composition of the leachates is unknown, targeted chemical analysis would be needed to identify the compounds inducing these effects. Furthermore, more experimental data is needed to compare dosing methods, using cells from different donors, to ensure that the obtained data is relevant for toxicological effects of MNPs occurring in humans.

## Supplementary Information

Below is the link to the electronic supplementary material.

Supplementary Material 1

Supplementary Material 2

Supplementary Material 3

Supplementary Material 4

## Data Availability

Raw data will be made available.

## Abbreviations

Abbreviation: Full name
ACTB: Beta actin
ALI: Air-Liquid-Interface
B2M: Beta-2-microglobulin
BSA: Bovine serum albumin
CCL2: Chemokine (C-C motif) ligand 2, or MCP-1
CXCL1: C-X-C motif chemokine ligand 1, or GROα
CXCL2: C-X-C motif chemokine ligand 2, or MIP2α
CXCL8: C-X-C motif chemokine ligand 8
GROα: Growth-regulated oncogene-alpha, or CXCL1
HBSS: Hank’s balanced Salt Solution
IL-8: Interleukin-8, or CXCL8
LDH: Lactate dehydrogenese
LDHmax: Maximum LDH response, LDH response of lysed cells
MCP-1: Monocyte chemotactic protein 1, of CCL2
MIP2α: Macrophage inflammatory protein 2-alpha, or CXCL2
MNPs: Micro- and nanoplastics
NHBEs: Normal Human Bronchial Epithelial cells
PA: Polyamide
PBS: Phosphate-buffered saline
PPIA: Cyclophilin A
PS: Polystyrene
PVC: Polyvinyl chloride
qPCR: Real-time quantitative PCR
RIVAES: Radial in vitro aerosol exposure system
RPL13A: Ribosomal protein L13a
sQCM: Quartz Crystal Microbalance
TEER: Transepithelial electrical resistance
