# Supplementary material for: Polyamide and polyvinyl chloride microplastics induce cytotoxicity and cytokine release in primary normal human bronchial epithelial cells
Source: Microplast nanoplast. 2026 May 19;6(1):50. doi: 10.1186/s43591-026-00200-w (PMC13350118; doi:10.1186/s43591-026-00200-w)
Supplement: Supplementary file 1 — Supplementary Material 1 [file 43591_2026_200_MOESM1_ESM.docx]

# Supplementary file 1:

# Comparison of exposure methods: nebulization and quasi-ALI exposures

For the direct comparison between exposure methods, a separate experiment was conducted, using cells from three different donors (see above).

Three conditions were tested, and for each a solvent control was included. Two inserts from each donor were exposed to each condition. The experimental design is shown in Table S1.1.

Table S1.1: Experimental design for comparison between exposure methods and the role of BSA

| **Condition ID** | **Exposure method** | **Condition** | **Type of MNP** | **Dose of MNP** |
| --- | --- | --- | --- | --- |
| A | Nebulization | PBS + 0.05% BSA + 1% 1-propanol | x | x |
| B | Nebulization | PBS + 0.05% BSA + 1% 1-propanol | PVC < 1 µm | 0.1ug/cm^2^ |
| C | Nebulization | no BSA + 100% 1-propanol | x | x |
| D | Nebulization | no BSA + 100% 1-propanol | PVC < 1 µm | 0.1ug/cm^2^ |
| E | Quasi-ALI | PBS + 0.05% BSA + 1% 1-propanol | x | x |
| F | Quasi-ALI | PBS +0.05% BSA + 1% 1-propanol | PVC < 1 µm | 0.1ug/cm^2^ |
| G | N/A | IC | x | x |

*x means that these cells did not receive a dose; N/A = not applicable, incubator controls (IC) were kept in the incubator.*

*For each condition, 2 inserts per donor were exposed.*

*Conditions A, C and E and 5 served as a control for conditions B, D, F respectively.*

*As MNPs were suspended in 1-propanol, the same quantity of 1-propanol was added to the control conditions. Conditions 3 and 4 are comparable to the nebulization experiments described above.*

Suspensions for comparison of the exposure method were prepared in two steps. First, a 10 times concentrated pre-dilution was made in 0.5% BSA (bovine serum albumin)/PBS or 1-propanol, after thoroughly vortexing the stocks. This pre-dilution was further diluted with PBS or 1-propanol to reach the desired PVC concentration (60 µg/mL for nebulization exposures, 5.6 µg/mL for quasi-ALI exposures). The applied dose for the quasi-ALI exposure corresponded to the highest dose of the nebulization exposures (0.1 µg/cm^2^), as can be calculated using the surface area of the transwell insert (1.12 cm^2^) and the applied droplet volume (20 µL for quasi-ALI exposure). For the suspensions containing BSA (A9418, Sigma, LOT: SLCK3989), the pre-dilution was prepared in 0.5% BSA/PBS to prevent particle agglomeration, resulting in 0.05% BSA in the final working suspension. Final 1-propanol concentrations were 100% (condition 4) or 1% (conditions 2 and 6). Proper solvent controls containing the same amount of BSA and/or 1-propanol were included for each donor (conditions 1, 3 and 5), as well as an unexposed incubator control.

## Results of the comparison between exposure methods

Exposure via nebulization was compared to exposure via quasi-ALI, using PVC <1µm particles and a dose of 0.1ug/cm^2^, as the exposure method may explain the differences in cytokine responses with previously published work (see discussion section). In addition to the exposure method, another important difference between the experiments was the addition of BSA for quasi-ALI exposure, to avoid particle aggregation in watery solutions. For the nebulization exposure, we therefore also included conditions with and without the addition of BSA.

Cytokine secretion was measured 24 hours after exposure. Similar to the experiments described above, only the levels of IL-8 and MCP-1 were found to be affected by the exposure (Figure S1.1). As cytokine responses were corrected for variation between donors, values are expressed as the ratio to the donor-matched average of quasi-ALI vehicle control group (G in Figures S1.1 and S1.2). The absolute data can be found in Supplementary file 4.


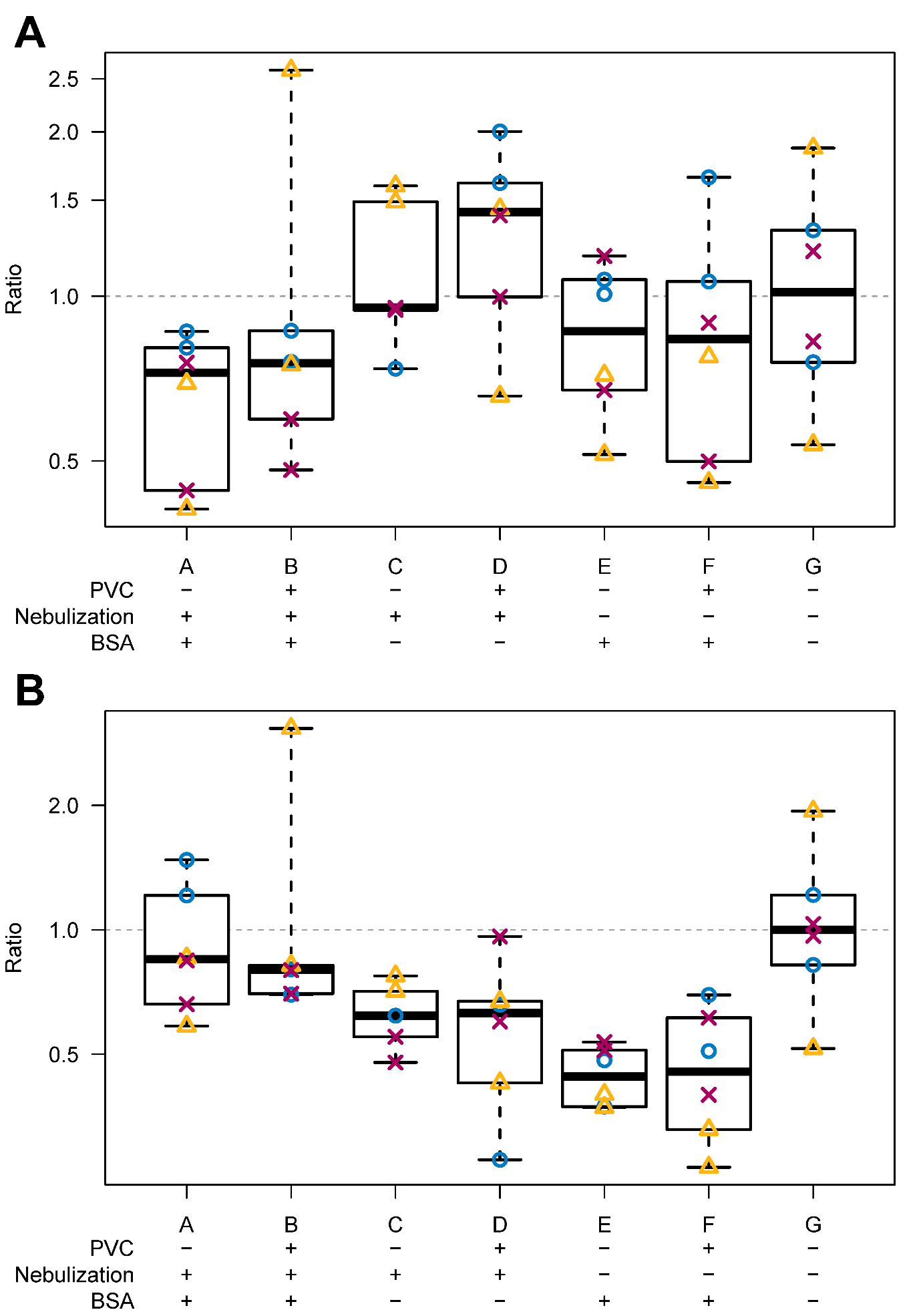


*Figure S1.1: IL-8 (A) and MCP-1 (B) release of NHBE cells of three donors exposed to PVC <1µm particles, either with or without BSA and using exposure by nebulization or applying the particles quasi-ALI on the apical side. Values are expressed as the ratio to the donor-matched average of quasi ALI vehicle controls (G). Colors indicate the three different donors. Two inserts per donor were used. The box indicates the interquartile range, whiskers indicate the maximum-minimum range, the line in the box indicates the median and the circle indicates the geometric mean. Statistical analysis is shown in Table 4.*

*Labels x-axis:*

*A: Vehicle control nebulization, with BSA, 1% propanol*

*B: PVC nebulization, with BSA, 1% propanol*

*C: Vehicle control nebulization, no BSA, 100% propanol*

*D: PVC nebulization, no BSA, 100% propanol*

*E: Vehicle control quasi ALI, with BSA, 1% propanol*

*F: PVC quasi ALI, with BSA, 1% propanol*

*G: Incubator control, no BSA*

At the end of the incubation period, cells were harvested for gene expression measurements of *CXCL1*, *CXCL2* and *CXCL8*. Results are shown in Figure 6.


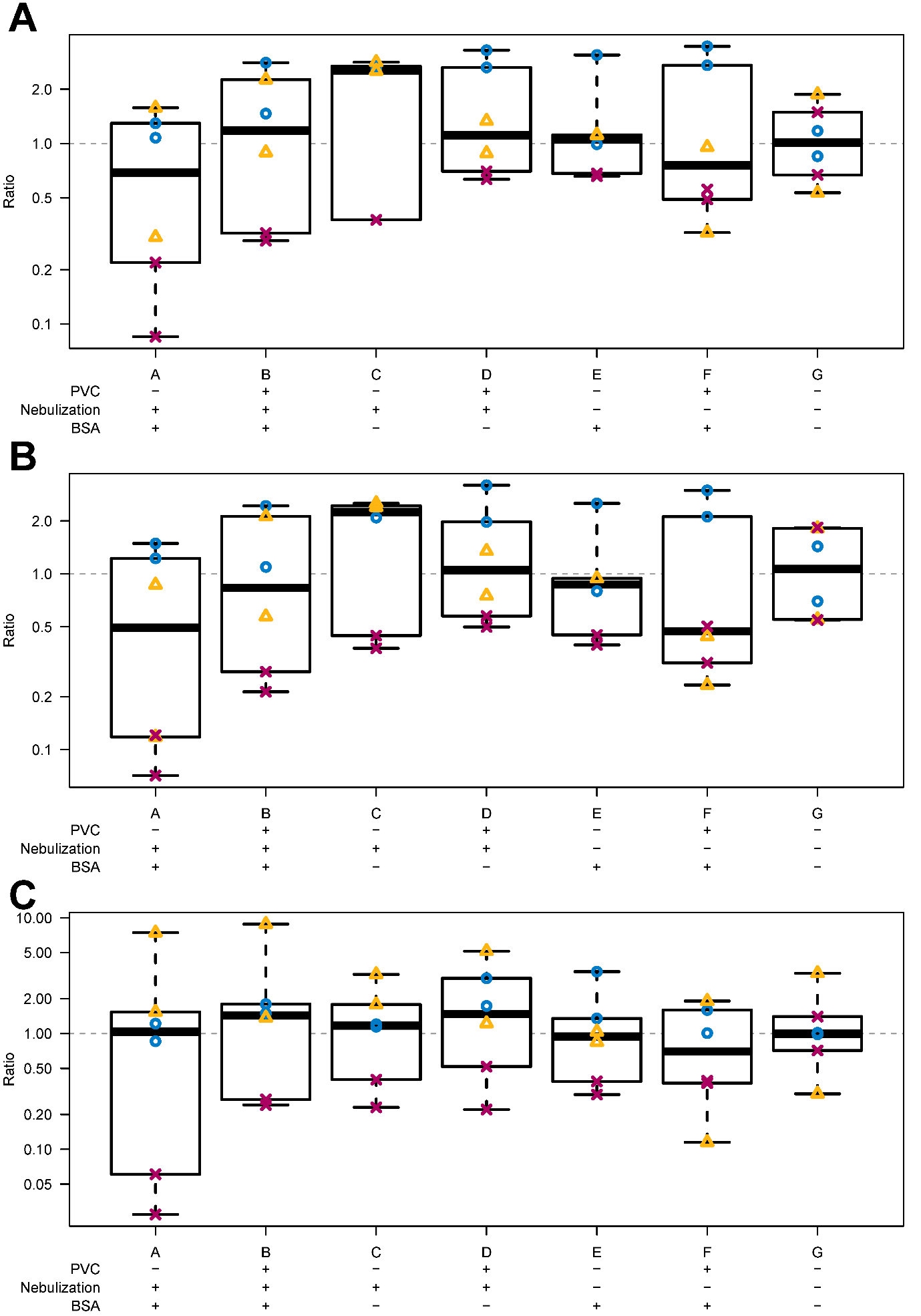


*Figure S1.2: CXCL1 (A), CXCL2 (B) and CXCL8 (C) expression by NHBE cells of three donors exposed to PVC <1µm particles, either with or without BSA and using exposure by nebulization or quasi-ALI. Values are expressed as the ratio to the donor-matched average of quasi ALI vehicle controls (G). Colors indicate the three different donors. Two inserts per donor were used. The box indicates the interquartile range, whiskers indicate the maximum-minimum range, the line in the box indicates the median and the circle indicates the geometric mean. Statistical analysis is shown in Table 4.*

*Labels x-axis:*

*A: Vehicle control nebulization, with BSA, 1% propanol*

*B: PVC nebulization, with BSA, 1% propanol*

*C: Vehicle control nebulization, no BSA, 100% propanol*

*D: PVC nebulization, no BSA, 100% propanol*

*E: Vehicle control quasi ALI, with BSA, 1% propanol*

*F: PVC quasi ALI, with BSA, 1% propanol*

*G: Incubator control, no BSA*

*Table S1.2****:*** *Relative contribution of the variable to the response measured, including statistical analysis for cytokine and gene expression data. Data were expressed at a log2-scale and analysed using a mixed-model ANOVA, with particle/vehicle exposure, nebulization/quasi-ALI and presence/absence of BSA as independent variables and donor as a covariable. Donor-related differences were all significant (all p-values < 0.007) but are not shown as they only served to correct other variables for donor variation.* *Significance is indicated with asterisks: * = p<0.05; ** = p<0.01*

|  | **marker** | **variable** | **estimate** | **std. error** | **p-value** | **significance** |
| --- | --- | --- | --- | --- | --- | --- |
| Cytokines | IL-8 | particle exposure vs no particle exposure | 0.23 | 0.19 | 0.24 |  |
|  |  | nebulization vs quasi-ALI | -0.04 | 0.19 | 0.85 |  |
|  |  | BSA present vs absent | -0.59 | 0.19 | 4.2E-03 | ** |
|  | MCP-1 | particle exposure vs no particle exposure | -0.26 | 0.23 | 0.28 |  |
|  |  | nebulization vs quasi-ALI | 0.40 | 0.23 | 0.10 |  |
|  |  | BSA present vs absent | -0.05 | 0.23 | 0.83 |  |
| Gene expression | CXCL1 | particle exposure vs no particle exposure | 0.34 | 0.28 | 0.23 |  |
|  |  | nebulization vs quasi-ALI | -0.26 | 0.28 | 0.37 |  |
|  |  | BSA present vs absent | -0.64 | 0.28 | 0.03 | * |
|  | CXCL2 | particle exposure vs no particle exposure | 0.30 | 0.31 | 0.34 |  |
|  |  | nebulization vs quasi-ALI | -0.29 | 0.31 | 0.37 |  |
|  |  | BSA present vs absent | -0.97 | 0.31 | 3.8E-03 | ** |
|  | CXCL8 | particle exposure vs no particle exposure | 0.31 | 0.43 | 0.47 |  |
|  |  | nebulization vs quasi-ALI | 0.03 | 0.43 | 0.95 |  |
|  |  | BSA present vs absent | -0.54 | 0.43 | 0.21 |  |

Most of the variation in the responses on both cytokine release and gene expression could be attributed to differences between donors. Particle exposure did not result in significant effects on the cells. Nevertheless, BSA resulted in a decrease of *CXCL1* and *CXCL2* expression and IL-8 release compared to the absence of BSA (in nebulization exposure, see Table S1.2). A lower expression of *CXCL1* and *CXCL2* by BSA (negative estimates in Table 4) may also be attributed to a higher expression by 1-propanol; e.g. when BSA was present, the 1-propanol concentration was 1% (not 100%) whereas the when BSA was absent, the 1-propanol concentration was 100%. However, with our experimental setup this difference could not be discriminated. Exposure by nebulization resulted in a decrease of MCP-1. No effect of the exposure to PVC was found, in contrast to the experiment with mixed donors, showing an increase in IL-8 and decrease of MCP-1 by PVC <1µm particles.

## Discussion of comparison between exposure methods

We assessed in a pilot experiment the importance of the exposure method and of the addition of BSA on the effects observed. The effect of the PVC particles on the cells was limited in this experiment compared to the results reported in the manuscript. This difference could potentially be explained by differences in sensitivity of the cells of the donors used. Most of the variation in the responses on both cytokine release and gene expression could be attributed to differences between donors, therefore, no definite conclusion can be drawn with respect to the exposure method. Nevertheless, BSA with 1% 1-propanol resulted in a decrease of *CXCL1* and *CXCL2* expression and IL-8 release compared to the absence of BSA and 1-propanol as carrier (nebulization exposure). A lower expression of *CXCL1* and *CXCL2* by BSA with 1% 1-propanol may also be attributed to a higher expression by 1-propanol.

However, a limitation of the experimental setup is that low concentration of propanol (1%) were always in the presence of BSA, whereas high concentration of propanol (100%) were always without BSA. This means that the effects of BSA could not be independently evaluated from the effects of 1-propanol. The comparison between nebulization and quasi-ALI could be made, as the applied suspension was the same (0.05% BSA and 1% 1-propanol), conditions A vs E and B vs F, but this did not result in a different outcome, likely due to the absence of the effects of PVC.

Therefore, further experimental data is needed to compare dosing methods and the role of BSA in particle suspensions on biological outcomes, to ensure that the obtained data is relevant for toxicological effects occurring in humans.
