# Supplementary material for: Polyamide and polyvinyl chloride microplastics induce cytotoxicity and cytokine release in primary normal human bronchial epithelial cells
Source: Microplast nanoplast. 2026 May 19;6(1):50. doi: 10.1186/s43591-026-00200-w (PMC13350118; doi:10.1186/s43591-026-00200-w)
Supplement: Supplementary file 2 — Supplementary Material 2 [file 43591_2026_200_MOESM2_ESM.docx]

# Supplementary file 2:

# Validation of the RIVAES

**Introduction**
The ‘Cloud’ system from Vitrocell is designed to expose lung cells, cultured in inserts, to airborne substances that are dissolved or suspended in water. The inserts are positioned on a plate heated to 37°C, over which a lid equipped with an ultrasonic nebulizer is placed. This nebulizer generates an aerosol from the test solution or suspension, allowing particles to settle gradually onto the inserts below.

Ideally, particle deposition should be uniform across all inserts. However, practical experience has shown considerable variability—sometimes up to several tens of percent difference between the highest and lowest dosed inserts. In some cases, deposition was found to be inversely proportional to the insert’s distance from the nebulizer.

To improve deposition uniformity, a new version of the Cloud system, termed radial *in vitro* aerosol exposure system (RIVAES), was developed. In RIVAES, inserts are positioned radially relative to the nebulizer, unlike the original Cloud 12 design, which uses a 3 x 3 grid. The radial configuration ensures equal distances between each insert and both the nebulizer and the chamber wall. In the original Cloud 12, the central insert is closest to the nebulizer and furthest from the wall.

This supplementary file describes the experiments conducted to compare deposition variability between the new RIVAES and the original Cloud 12 designs.

**System specifications**

- **Cloud 12:** The original Vitrocell system with inserts and a quartz crystal microbalance (QCM) arranged in a 3 x 3 grid, covering a total surface area of 141.6 cm². The rectangular chamber is made of plastic with an internal volume of 2172 cm³.
- **RIVAES:** The improved, radial design with inserts placed equidistantly on a 9 cm diameter circle, total area 120.8 cm². The chamber consists of a glass cylinder with a PMMA lid, with an internal volume of 1570 cm³.

*Table S2.1: Specifications of both systems.*

|  | System | |  |
| --- | --- | --- | --- |
|  | Cloud 12 | RIVAES |  |
| length | 11.3 |  | cm |
| width | 12.4 |  | cm |
| diameter |  | 12.4 | cm |
| A_ground_ | 140.12 | 120.8 | cm^2^ |
| height | 15.5 | 13.0 | cm |
| volume | 2172 | 1570 | cm^3^ |
| int surface hood | 875 | 627 | cm^2^ |
| A_hood_/A_ground_ | 6.2 | 5.2 |  |

Figure S2.1: Location of the wells and QCM in the Cloud 12 (left) and RIVAES (right)

**Additional Materials and Equipment**

- 12W inserts (Corning Costar 12W)
- Quartz Crystal Microbalance (Vitrocell)
- PBS, physiological saline, ultrapure water (MQ)
- Ultrasonic nebulizer (Aeroneb, 4.0–6.0 µm)
- 200 µL pipette

**System Requirements**
The variability in particle deposition across inserts in RIVAES must be equal to or less than that observed in Cloud 12.

**Methods**
Four test substances were nebulized in triplicate in both Cloud 12 and RIVAES, using the same QCM for all measurements:

- Sodium fluorescein (30 µg/mL, 1:1 in PBS)
- Polystyrene latex (PSL) 2 µm (25 mg/mL, 1:1 in MQ, 1/100 physiological saline)
- Polystyrene latex 0.1 µm (same as above)
- TiO₂ (500 µg/mL, sonicated)

The nebulization procedure followed standard RIVM protocols:

1. Rinse nebulizer with ultrapure water
2. Place inserts in Cloud, add 0.3 mL PBS per insert
3. Confirm QCM stability
4. Nebulize 200 µL test solution
5. Wait 6 minutes
6. Remove and dry chamber lid
7. After 1 minute, replace lid
8. Wait 3 minutes
9. Sample insert contents for analysis

Sodium fluorescein and polystyrene latex samples were analyzed using an ELISA reader; TiO₂ samples were analyzed by ICP-MS. QCM deposition was recorded for each run.

**Results**

For each run, the variability in particle deposition among the different inserts was calculated. From these data, the mean insert-to-insert variability was determined. Additionally, for each substance, the maximum possible deposition on the QCM was calculated, taking into account the amount of non-volatile material nebulized per run and the total surface area of the Cloud system. The nebulization efficiency was then calculated as the ratio of the average deposition measured by the QCM to the maximum theoretical deposition.

Detailed results and calculations, including maximal deposition and measured deposition by QCM and fluorescence, are available upon request. The key results per substance (averaged over three runs) are summarized In tables S2.2-S2.5.

Table S2.2: deposition results of Sodium fluorescein in Cloud 12 and RIVAES

| Sodium fluorescine | Cloud 12 | RIVAES |
| --- | --- | --- |
| mean insert to insert variability | 10.6% | 5.8% |
| Expected max deposition (ng/cm2) | 15116 | 17539 |
| mean deposition QCM | 7165 | 11931 |
| 2 SEM | 1257 | 516 |
| deposition efficiency QCM | 47.4% | 68.0% |

Table S2.3: deposition results of PSL 0.1µm in Cloud 12 and RIVAES

| PSL 0.1 µm | Cloud 12 | RIVAES |
| --- | --- | --- |
| mean insert to insert variability | 1.7% | 1.5% |
| Expected max deposition (ng/cm2) | 17792 | 20644 |
| mean deposition QCM | 13622 | 20213 |
| 2 SEM | 1958 | 2458 |
| deposition efficiency QCM | 76.6% | 97.9% |

Table S2.4: deposition results of PSL 2µm in Cloud 12 and RIVAES

| PSL 2 µm | Cloud 12 | RIVAES |
| --- | --- | --- |
| mean insert to insert variability | 1.3% | 0.7% |
| Expected max deposition (ng/cm2) | 17792 | 20644 |
| mean deposition QCM | 9581 | 11008 |
| 2 SEM | 243 | 1993 |
| deposition efficiency QCM | 53.9% | 53.3% |

Table S2.5: deposition results of TiO_2_ in Cloud 12 and RIVAES

| TiO_2_ | Cloud 12 | RIVAES |
| --- | --- | --- |
| mean insert to insert variability | - * | -* |
| Expected max deposition (ng/cm2) | 714 | 828 |
| mean deposition QCM | 382 | 509 |
| 2 SEM | 124 | 175 |
| deposition efficiency QCM | 54% | 61% |

* The Ti concentrations measured by ICP-MS were very low. Samples were stored for approximately two months before analysis; partial evaporation of aqua regia and/or Ti adhering to the plate walls may have occurred.

**Conclusion**

The variability in particle deposition among the inserts in RIVAES was lower for all tested substances compared to Cloud 12, fulfilling the predefined requirement for the system.
