# Supplementary material for: Polyamide and polyvinyl chloride microplastics induce cytotoxicity and cytokine release in primary normal human bronchial epithelial cells
Source: Microplast nanoplast. 2026 May 19;6(1):50. doi: 10.1186/s43591-026-00200-w (PMC13350118; doi:10.1186/s43591-026-00200-w)
Supplement: Supplementary file 3 — Supplementary Material 3 [file 43591_2026_200_MOESM3_ESM.docx]

# Supplementary file 3: Particle size distributions before and after nebulization


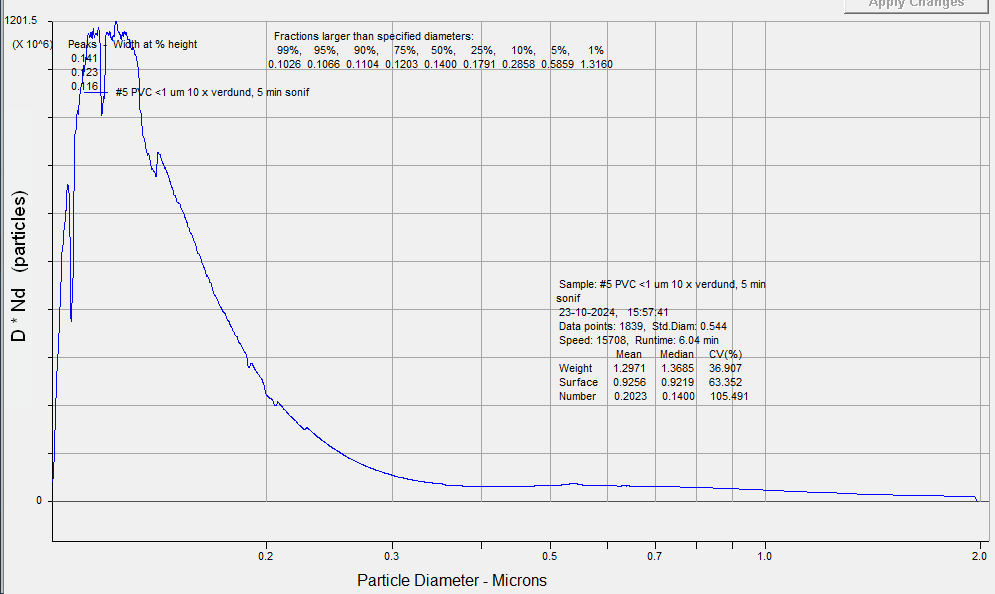


*Figure S3.1: PVC <1 µm before nebulization*


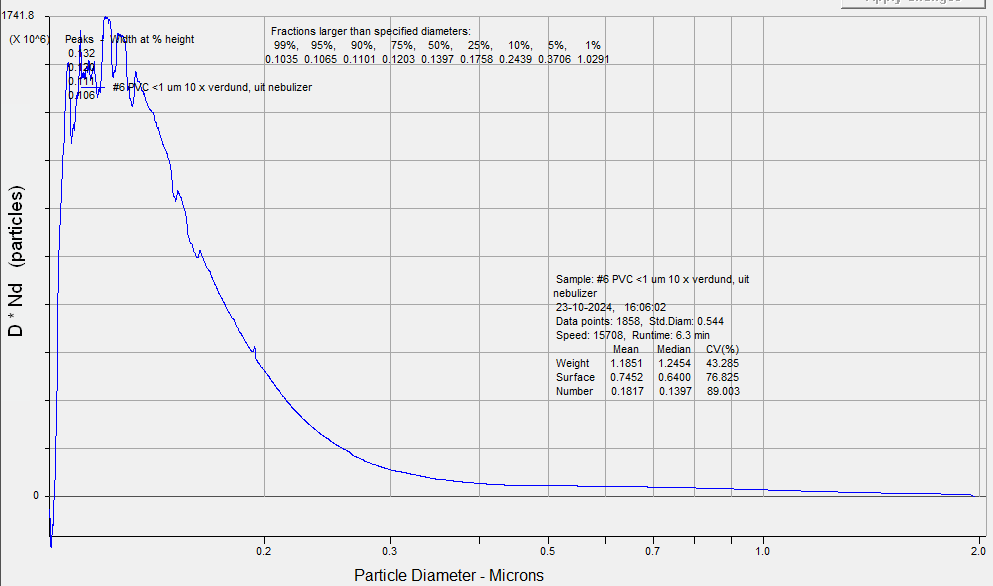


*Figure S3.2: PVC <1 µm after nebulization*


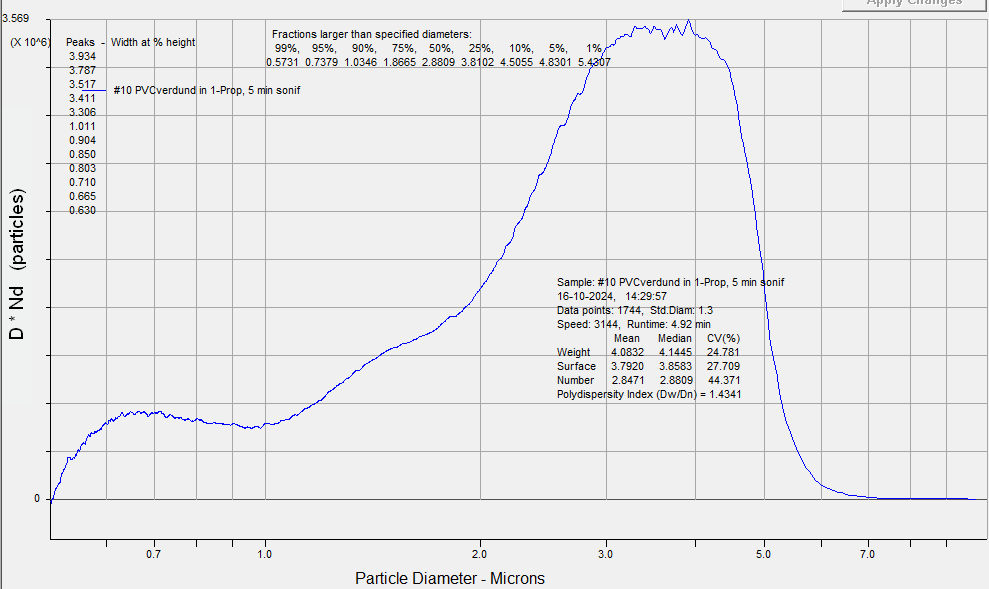


*Figure S3.3: PVC 1-5 µm before nebulization*


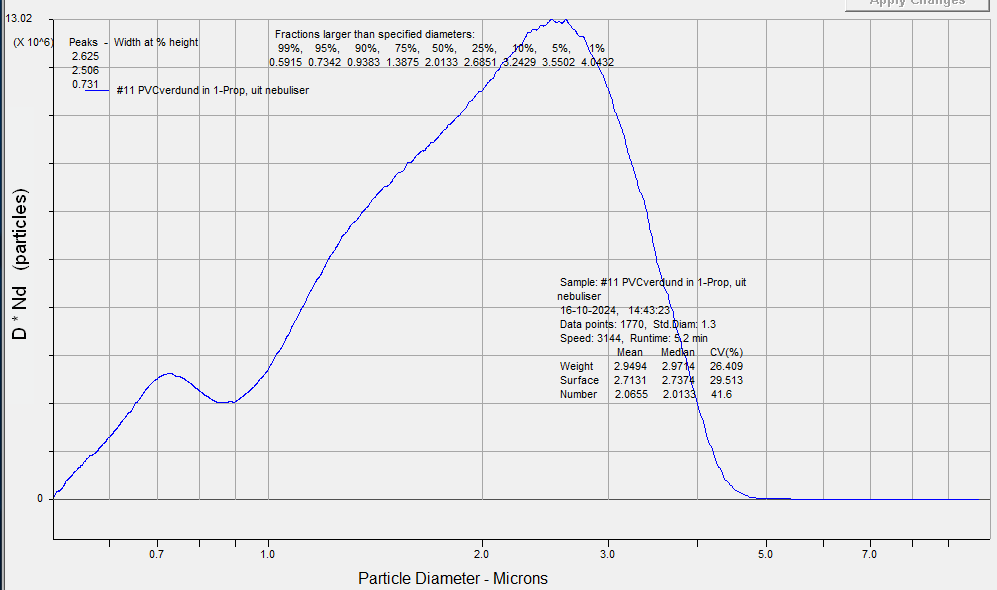


*Figure S3.4: PVC 1-5 µm after nebulization*


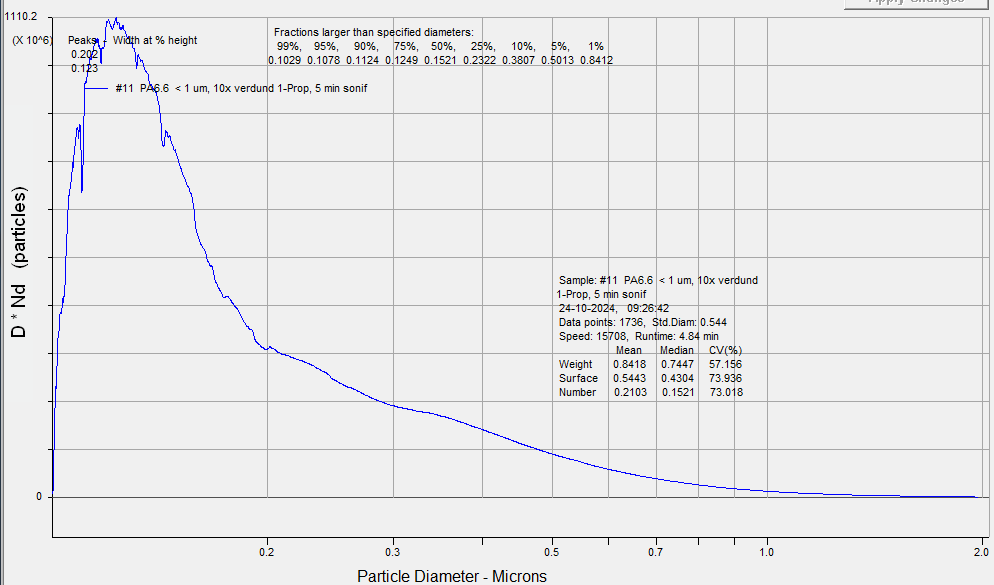


*Figure S3.5: PA <1 µm before nebulization*


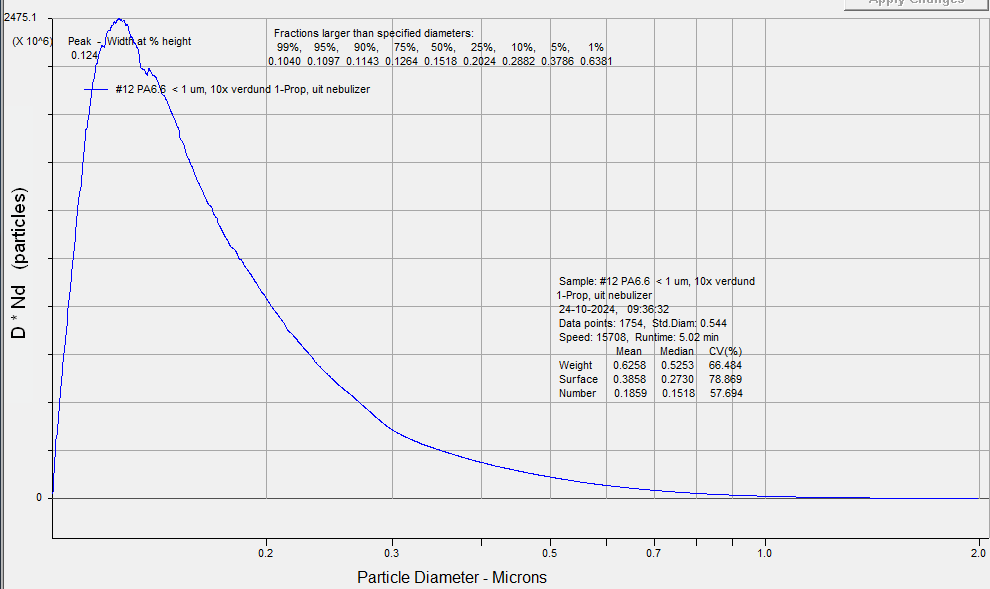


*Figure S3.6: PA <1 µm after nebulization*


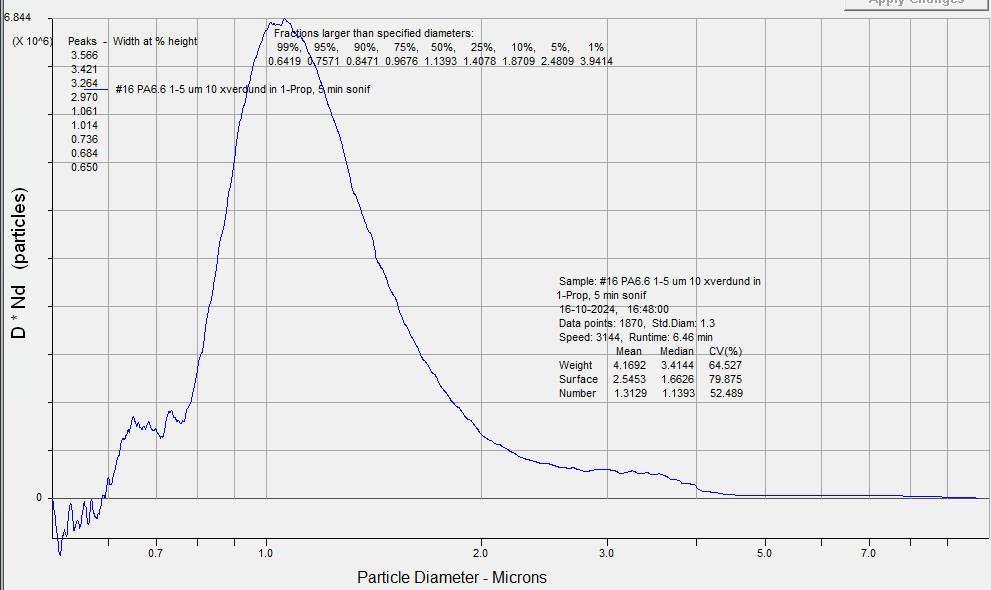


*Figure S3.7: PA 1-5 µm before nebulization*


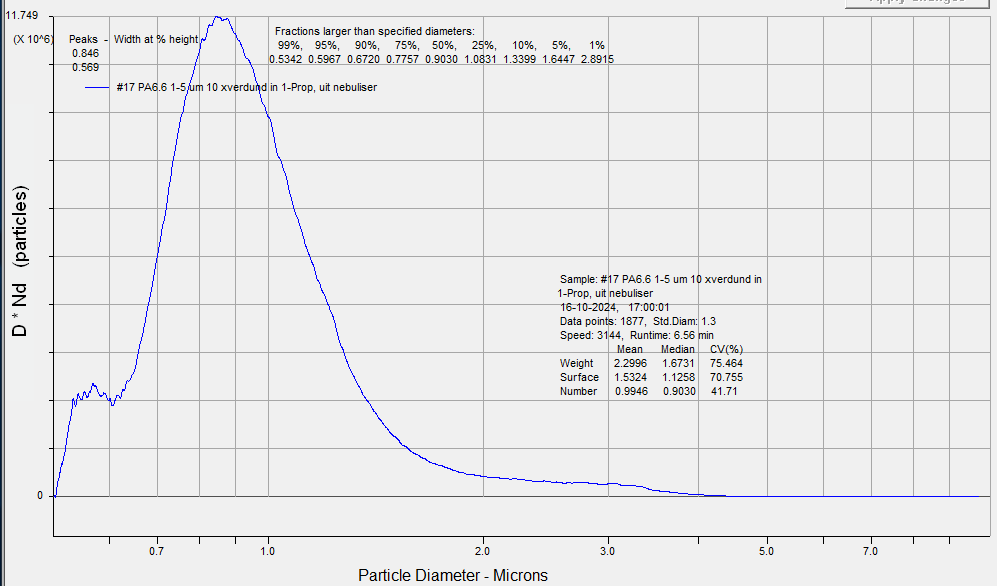


*Figure S3.8: PA 1-5 µm after nebulization*


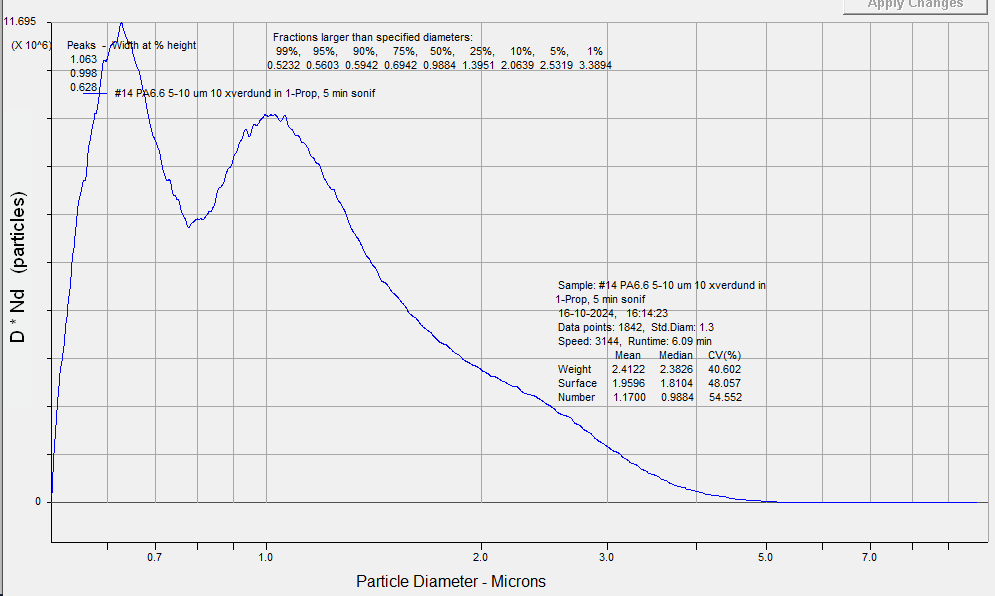


*Figure S5.1: PA 5-10 µm before nebulization*


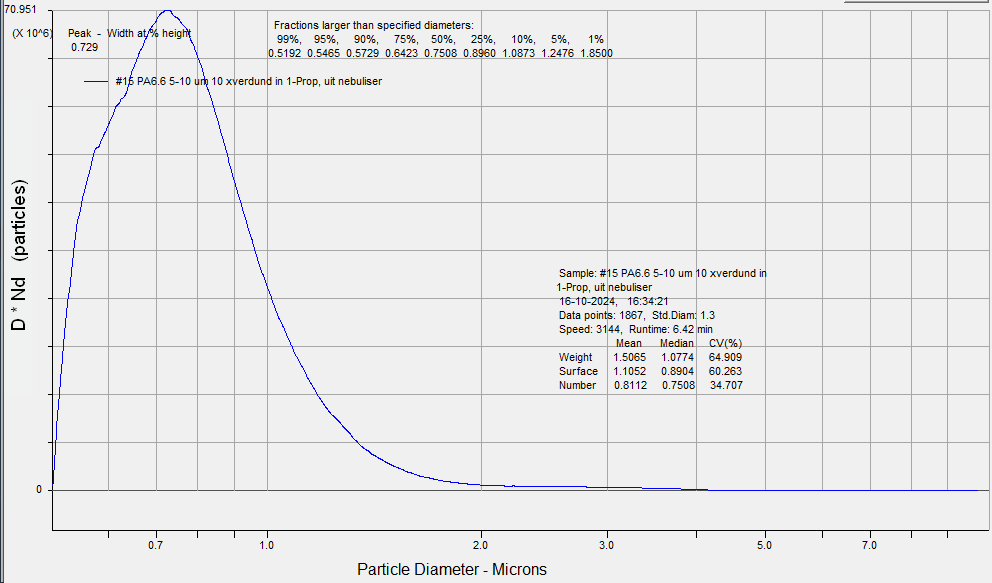


*Figure S5.2: PA 5-10 µm after nebulization*
